# Facts Concerning the Efficacy of Alkalis in Diseases of the Alimentary Canal. Communicated in a Letter from Mr. Joseph Bringhurst, Jun. to Dr. Miller

**Published:** 1802-10-01

**Authors:** 


*Facts concerning the Efficacy of Alkalis in Diseases of the Alimentary Canal. Communicated in a Letter from* Mr. JOSEPH BRINGHURST, jun. *to* Dr. MILLER.

IN glancing over a page of the laſt number of the Medical Repoſitory, my attention was arreſted by an account of accidental cures of the diſeaſe termed Bilious Colic, by means of oils and alkalis. I can furniſh additional evidence of the benefit of alkalis in that diſeaſe, and in thoſe which ariſe from the ſame cauſe.

In the year 1793, I was grievouſly afflicted by dyſpepſia, and frequently attacked by violent colic. As I was particularly attentive to my diet, I informed my phyſician, that I had obſerved thoſe attacks of colic often followed an acid taſte in my mouth, and were uniformly produced from my eating the ſmalleſt portion of pickle. Theſe circumſtances led him to a ſucceſsful mode of practice in my diſeaſe. He combined one drachm of pure pot-aſh with four ounces of water and a few drops of peppermint oil. Of this ſolution he directed me to take a tea-ſpoon full every ten minutes, until I obtained relief. I complied with his direction, and always found eaſe in a few minutes after I had taken the alkali. A friend, who laboured under dyſpepſia, informed me that he always found bread containing pot-aſh more ſuitable than any other for his ſtomach.

I ſhould premiſe that I am an Apothecary, and conſequently all my practice has been among poor people, who could not afford to pay a phyſician. A poor woman, during ſeveral weeks, laboured under a fever and ague. She became ſo much enfeebled that ſhe could ſcarcely walk. Her aſpect was cadaverous. She complained of great weight about her heart, and a ſenſe of ſuffocation. Peruvian bark irritated her ſtomach. I ſuſpected a vitiation of the fluids of her ſtomach, and conjectured that acidity was the chief cauſe of her ſenſations. One quarter of an ounce of magneſia, taken daily for three days, relieved her from the weight and ſenſe of ſuffocation, and then bark completed her cure. In fever and ague I always combine the alkali, vulgarly called ſal. tart. with bark. One ounce of bark, and half a drachm of the alkali, frequently cure perſons in two or three days.

About ſix weeks ſince, a man of this borough ſent his wife with a requeſt that I would call to ſee him. I complied with his deſire, and found him very ill. He informed me that he had ſent for me with the hope of receiving benefit from my advice. I obſerved, in reply, that I was not a phyſician, and as he was very ill, his diſeaſe required a perſon of more ſkill than myfelf. He urged me to prefcribe for him—in truth, he almoft compelled me to do fo. He informed me that he had been ill for feveral months, and that, during the fix laft weeks, he had been wearing away with a violent flux. Large quantities of blood came from him two or three times daily, and he was obliged to ufe the clofe-ftool from ten to forty times during each twenty-four hours. His evacuations of blood were always preceded by intenfe and cutting pains in his abdomen, and fenfations which impreffed him with fears of his hip bones-tearing afunder. From the colour and fœtor of his evacuations, and the cutting fenfations of which he complained, I fufpected a daily generation of acid in his ftomach and inteftines, and therefore refolved to adminifter alkalies. I immediately combined two ounces of pure foda with thirty-two grains of ipecacuanha. I divided the whole into fixteen dofes, and ordered the patient to take three daily. I combined the ipecacuanha with the hope that it would float on the fluids of his ftomach; and after the foda had united with the acid of his ftomach, and formed a purgative falt, the powder would follow it, and operate as a tonic on his ftomach and inteftines.—The man is now in perfect health. When he had taken one ounce of the foda thus combined, his ftools were diminifhed in number, quantity and appearance. They began to affume a natural colour, and were free from blood. In ten days he was able to walk about, and in lefs than three weeks he thought himfelf well. I charged him to eat fimple food, and avoid fuch meats as would opprefs his ftomach. He foolifhly tranfgreffed, by eating faufages fried in lard, and brought on his pains and bloody ftools. Three dofes of foda relieved him; and, by care in his diet, he is now in perfect health.

